# Understanding Sensory Information Processing Through Simultaneous Multi-area Population Recordings

**DOI:** 10.3389/fncir.2018.00115

**Published:** 2019-01-09

**Authors:** Elizabeth Zavitz, Nicholas S. C. Price

**Affiliations:** ^1^Department of Physiology, Biomedicine Discovery Institute, Monash University, Clayton, VIC, Australia; ^2^Centre of Excellence for Integrative Brain Function, Monash University Node, Clayton, VIC, Australia

**Keywords:** neuronal populations, hierarchical processing, neural computation, sensory coding, inter-area communication

## Abstract

The goal of sensory neuroscience is to understand how the brain creates its myriad of representations of the world, and uses these representations to produce perception and behavior. Circuits of neurons in spatially segregated regions of brain tissue have distinct functional specializations, and these regions are connected to form a functional processing hierarchy. Advances in technology for recording neuronal activity from multiple sites in multiple cortical areas mean that we are now able to collect data that reflects how information is transformed within and between connected members of this hierarchy. This advance is an important step in understanding the brain because, after the sensory organs have transduced a physical signal, every processing stage takes the activity of other neurons as its input, not measurements of the physical world. However, as we explore the potential of studying how populations of neurons in multiple areas respond in concert, we must also expand both the analytical tools that we use to make sense of these data and the scope of the theories that we attempt to define. In this article, we present an overview of some of the most promising analytical approaches for making inferences from population recordings in multiple brain areas, such as dimensionality reduction and measuring changes in correlated variability, and examine how they may be used to address longstanding questions in sensory neuroscience.

## Introduction

The cortex contains a multitude of representations of sensory information that are anatomically segregated by sensory modality (e.g., somatosensory vs. auditory), and by specialty within a modality (e.g., visual motion vs. visual form). Following recent advances in technology, large-scale recordings of neuronal population activity now extend across the boundaries of cortical areas. This presents an opportunity to understand the nature of inter-area neural processing. Many inter-neuronal and inter-area phenomena exist on timescales of milliseconds. In order to characterize this short-timescale activity requires electrophysiological approaches, which allow action potentials and local field potentials (LFPs) to be recorded. Although the largest simultaneous recordings of the functional activity of neuronal ensembles are now conducted with cellular-resolution imaging, and while cell-type specific genetic promoters promise recordings from neurons with known classes (Luo et al., 2008), in this article we will focus on experiments involving extracellular electrophysiological measurements, because these afford the temporal resolution required to address the analytical questions we pose. We mainly consider cortico-cortical processing in non-human primates, but these advances are complemented by substantial work in other species, and involving sub-cortical areas, which will be necessary to bridge the gap between understanding circuit architecture and large-scale network dynamics. Cortico-cortical processing is a good first frontier in multi-area population analysis as cortical architecture is well-characterized and similar between brain areas. Further, we mainly consider questions pertinent to data sets with population recordings from multiple brain areas simultaneously, but draw inspiration from analytical methods applied to either population recordings from one brain area, or recordings of two units in different areas.

## Why and How Should We Make Simultaneous Multi-Area Population Recordings?

The transition from recording from a single site at one time to recording population activity was a meaningful one for systems electrophysiology (Brown et al., 2004; Yuste, 2015). Recording from populations allows us to “embrace single-neuron heterogeneity” (Cunningham and Yu, 2014), and reveals structure in the signals across multiple neurons that we would not be able to recover any other way, such as their correlated variability (Zavitz et al., 2016; Bondy et al., 2018), and how population representations change within a subspace over time or depending on context (Churchland et al., 2012). Recording simultaneously from two or more neurons has advanced theories relating to how different types of “noise,” or inter-trial variability, affect stimulus discrimination (Zohary et al., 1994; Shadlen and Newsome, 1998; Cohen and Kohn, 2011; Kohn et al., 2016), and how decisions are generated based on the accumulation of evidence (Yates et al., 2017).

**Figure 1:**
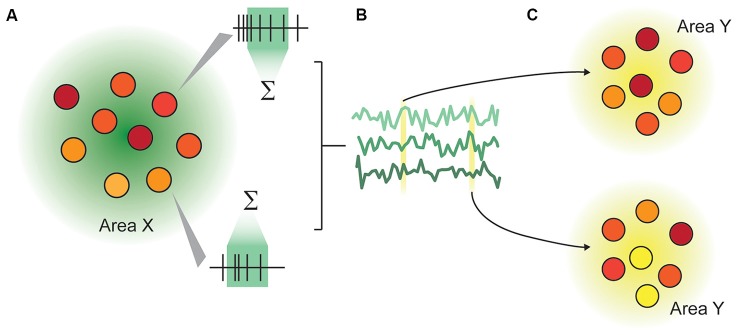
Illustration of the representation-communication framework for neuroscientific questions. **(A)** A pattern of activity within an area or population of neurons is captured as firing rates over a specified time window. In this rendering, each circle represents a neuron, and the color represents that neuron’s instantaneous activity, which continually changes over time. We measure representations not in an instant, but typically in a rate code, by integrating spiking activity over a time window ranging from tens to hundreds of milliseconds. **(B)** The rates of n neurons in Area X are collectively a multidimensional “representation” that varies over time. This representation may be as concrete as the joint firing rates across the population, or may be abstracted through dimensionality reduction. **(C)** An area X may be inferred to communicate with area Y if the representation within area X modulates the representation in area Y in a systematic way.

Recording from multiple areas can reveal temporal correlations between the two areas (Wong et al., 2016), giving insight into inter-area connectivity. Beyond this, by making simultaneous multi-area population recordings, we are able to make inferences about how population representations in one area influence the representations in another on a trial-by-trial basis (Zandvakili and Kohn, 2015), and how inter-area communication changes depending on external factors such as attention (Ruff and Cohen, 2016). Multi-area population recordings are thus able to address two classes of questions: how representations are changed between cortical areas, and how communication is facilitated (Figure 1). Here, representations are defined as the structure of neuronal activity in an ensemble, and communication as a recoding process (Pitkow and Angelaki, 2017), in which the representation of information within the recipient area is measurably changed. A similar architecture is outlined in Fries (2015).

Most sensory neuroscience is predicated on developing an understanding of how a physical stimulus produces an observed neuronal response. However, beyond the level of our sensory receptors, neurons do not directly respond to sensory stimuli. Rather, they change their membrane potential and generate action potentials in response to precise patterns of inputs, received from a population of synaptically-connected neurons. By recording from connected brain areas, we can use the recordings from the source area to gain a better understanding of the true inputs to the recipient brain area, and how they are transformed in the downstream area.

## Promising Analytical Approaches

There are three major classes of analyses that have allowed researchers to draw novel conclusions about information processing between simultaneously recorded areas: lower-dimensional representations; pairwise correlated variability (“noise” correlations or “correlation structure”); and measures of spike-timing precision. The most valuable observations we derive from these analyses are often not their immediate outputs, but instead how these outputs change depending on other contextual variables such as the stimulus, behavior, or cognitive state.

### Lower-Dimensional Representations

**Figure 2:**
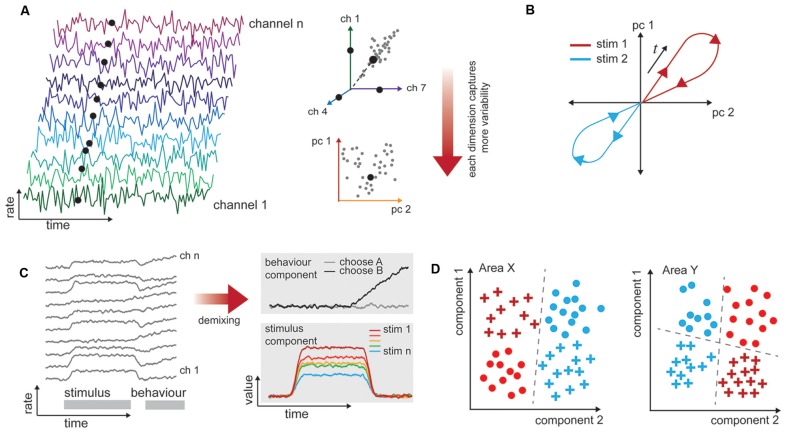
Procedures for analyzing high-dimensional neural data in a biologically informative way. **(A)** Illustration of dimensionality in multichannel recordings. Time-varying data are collected simultaneously from populations of neurons. These are typically spiking rates over some time window. The rates exist in a space that has the same dimensionality as the number of channels recorded. However, neuronal responses are typically not unique or independent, so it is likely that pairs of neurons have correlated firing rates (here, channels 1 and 7). This allows for dimensionality techniques (here, principal components analysis) to capture most of the variability in a reduced number of dimensions. **(B)** Population response trajectories to different stimulus conditions can be traced through this reduced space over time. **(C)** Firing rates of neurons, left, often relate to more than one experimental variable (here, stimulus and behavior, gray bars). The high-dimensional responses of many neurons may be reduced with supervision so that they are also de-mixed, and the independent stimulus and behavior selective responses are clear. **(D)** One-way representations change between brain areas is that they allow different variables to become more easily, or linearly, separable. In this example, one stimulus attribute is separable in Area X (color), while both shape and color are separable in Area Y, depending on the decision line (dashed).

Across a population of neurons, there is both diversity and redundancy in neuronal responses, and it can be difficult to gain any understanding of how sensory information is represented when the number of dimensions describing the data equals the observed number of neurons (Figure 2A). Dimensionality reduction techniques such as principal components analysis allow covariation between neurons to be collapsed (Figure 2B), and the resulting visualization can show how population representations shift as a function of time and stimulus properties (Figure 2C). By translating data into a reduced format, we can form intuitions and hypotheses about what would otherwise be an intractably large data set that may bear little relationship to stimulus variables at first examination (Cunningham and Yu, 2014). In this “state space” the aggregate population activity at any point in time may be represented by a single point (Figure 2B). This style of representation permits comparison across stimulus or behavioral characteristics independently of the often heterogeneous and complex selectivity of the neurons (as in Churchland et al., 2012; Mante et al., 2013). Dimensionality reduction can be achieved in a number of ways (principal components analysis, factor analysis, Gaussian process factor analysis, among others), with different methodological advantages but similar outcome: a reduced space in which to consider the variability of neuronal responses. Traditionally, the focus is on how this variability relates to the stimulus or behavior. With multi-area recordings, it is also appropriate to consider how the variability of neuronal responses in one area relates to the responses of a connected population.

In a typical experiment in which multiple factors can vary (e.g., stimulus value, animal behavioral state, motor outcome), the variability in neuronal responses across trials of the same type is the most interesting to the experimenter. Unsupervised approaches will operate on the data blind to these experimental manipulations or outcomes, and the components they extract may not isolate the impact of experimental variables of interest (Kobak et al., 2016). To address this shortcoming, a layer of supervision can be added to isolate experimental variables, e.g., hierarchical decomposition (Repucci et al., 2001; Maddess et al., 2006), demixed PCA (Kobak et al., 2016), and tensor component analysis (Williams et al., 2017). This means that the recovered components are those that best explain individual and paired factors of interest (Brendel et al., 2011; Kobak et al., 2016). We illustrate a simplified account of mixed “stimulus” and “behavior” signals in a population, and how these components may appear once demixed in Figure 2D. Although poorly explored thus far, we anticipate that this approach will be particularly valuable for analyzing multi-area data sets, because it will enable quantification of how the representations change together on a trial-by-trial basis.

Dimensionality reduction works by collapsing across shared variability that arises from variations in both the “signal” (i.e., tuning similarities) and the “noise” (i.e., trial-by-trial variations in responses to the same signal). To learn more about the nature of population representations and inter-area communication, we can examine the noise correlations in isolation.

### Noise Correlations

The spiking activity of neurons varies from trial to trial, even under identical stimulation conditions. In pairs of simultaneously recorded neurons, this variability tends to be shared: if one neuron fires at an above-average rate, others are likely to as well (Zohary et al., 1994). Because this shared variability is not related to the stimulus or signal, it is termed “noise” or “spike-count” correlations, and is quantified by the Pearson’s correlation coefficient between the spike counts of the two cells across repetitions of the same stimulus (Cohen and Kohn, 2011). The strength of the measured correlation depends on a number of factors, including the two neurons’ mean firing rate (de la Rocha et al., 2007), separation in cortical tissue (Smith and Kohn, 2008; Solomon et al., 2015; Rosenbaum et al., 2017), and similarity in tuning properties (Kohn and Smith, 2005).

The pattern of spike-count correlations we are able to observe can reflect global modulations in activity that affect the whole population (Goris et al., 2014) or synaptic architecture, which can describe either structural architecture like connectivity patterns (Hu et al., 2012) or functional architecture like moment-to-moment connectivity (Haider and McCormick, 2009). Functional architecture, and spike-count correlations, are changed by recruiting (Snyder et al., 2014) or adapting (Zavitz et al., 2016) different subpopulations of neurons. The magnitude and structure of pairwise correlated variability across populations of neurons relates to behavior (Gutnisky et al., 2017; Ni et al., 2018), how well stimulus parameters are represented (Moreno-Bote et al., 2014; Kohn et al., 2016; Zylberberg et al., 2016; Zavitz et al., 2017), and reflects the task the animal is performing (Bondy et al., 2018).

To measure spike-count correlations, spikes are typically counted in bins with sizes ranging from tens of milliseconds to one or two seconds. However, information is also present in the precise timing of spikes from a neuron, either relative to the LFP or the timing of spikes from other neurons. While longer bins increase the overall spike count and the reliability of the measure, the behavioral relevance of these timescales is not clear.

### Spike-Timing Precision

The precise timing relationships in the activity of groups of neurons, measured as synchrony or coherence, can inform us about coordinated spiking activity and communication (Jia et al., 2013; Zandvakili and Kohn, 2015). Synchronized firing across a diverse group of neurons may be an important way to encode complex stimuli (Singer et al., 1997), and pairs of neurons can coordinate firing at timescales as short as 1 ms (Palm et al., 1988). There is evidence that different information is encoded in spikes aligned with different phases of specific frequencies in the LFP (Womelsdorf et al., 2012; Wong et al., 2016) and neural activity with precise delays between populations of neurons and across cortical layers may even be critical to the process of information transmission (Bastos et al., 2015).

Spiking synchrony may be measured with a cross-correlogram—correlations in instantaneous spiking between neurons at a range of time delays. While spiking activity is best understood as a point-process in the time domain, the LFP is a continuous process in the time-frequency domain, characterized in terms of how the power and phase across different frequency bands change over time. A common way of relating these discrete and continuous processes is coherence, a frequency-dependent measure of signal correlation, that may be examined between spikes and the LFP recorded on the same or different electrodes (Jarvis and Mitra, 2001). These measures have been used to understand how pairs of neurons communicate within (Dean et al., 2012; Hagan et al., 2012) and between (Jia et al., 2013; Wong et al., 2016) cortical areas. Although their use has not yet been expanded to large-scale recordings, given that spikes are commonly described as the outputs of a neuron and the LFP represents the net synaptic input to the region near the electrode, these approaches correlating spiking and the LFP are some of the most direct for examining how communication occurs across area boundaries. There are not any widely adopted population measures of timing precision, and this presents a fruitful area for future development. The process of identifying assemblies of neurons that fire in concert (Singer et al., 1997) could be expanded to include more detailed temporal characterization.

## Viable Avenues of Inquiry

### How Do Brain Areas Communicate With One Another?

Information is flexibly and efficiently routed throughout the brain. Here, we define communication as signal propagation that produces a change in the representation by a recipient area. Part of the challenge for achieving inter-area communication is related to signal transmission: a signal must be able propagate reliably throughout the system without excessive attenuation or amplification (Shadlen and Newsome, 1998; Joglekar et al., 2018; van Vugt et al., 2018). This relies on inter-area anatomical connections as well as the network structure within an area (Joglekar et al., 2018). However, there is substantial evidence that successful inter-area communication also requires physiological coordination on millisecond time-scales (Fries, 2005, 2015).

Inter-area information transmission has been assessed using coherence measures across the V1-V2 boundary (Jia et al., 2013), and by the likelihood of spikes in a recipient area given the state of a source area (Zandvakili and Kohn, 2015). The quality of signal transmission has been measured by the number of spikes elicited in the recipient area following of electrostimulation of the source area (Ruff and Cohen, 2016). These approaches demonstrate an effect of state on a recipient area, or propagation, but they do not demonstrate that communication has occurred. This could be achieved with an additional analysis demonstrating improvement in coding in the recipient brain area. This may be done directly by assessing perception in an awake behaving animal or decoding the spiking activity in the anesthetized preparation; or indirectly by measuring representations or spike-count correlations. These early studies had a small number of electrodes in the recipient area, so such analyses would have been limited, but will be increasingly possible as recording capabilities improve. Changes in noise correlations between areas can also be interpreted as changes in the communication efficacy between areas. If correlations between areas increase, they share more trial-to-trial variability, which means signal transmission is enhanced, but it is unknown whether this also enhances the representation in the recipient brain area.

Within a single brain area, inferences may be made about the relationship between cortical state and coding efficacy by conditioning the data, or sorting population activity into states based on a variable of interest (e.g., up and down states based on firing rate; Arandia-Romero et al., 2016; Gutnisky et al., 2017), or behavioral outcome or strategy (Gilad et al., 2018). Recent work adapts this approach to two connected populations of neurons by estimating how the state of one area impacts coding in a recipient area, demonstrating how we might test the efficacy of neural communication more directly (Palmigiano et al., 2017). In simulations, they measured the relative phase of gamma bursts in two areas, and condition based on which area is leading. This enabled them to show that spiking activity in the leading area predicts spiking activity in the following area, suggesting that gamma bursts produce states that are conducive to spike transmission. However, the results of conditioning data should be interpreted with caution, as the variable chosen for conditioning will have multiple covariates.

### How Are Representations Transformed Between Areas?

Understanding population responses in terms of a low-dimensional representation has provided traction especially in our understanding of how neurons with complex selectivity represent stimuli and guide behavior. In the context of multi-area recordings, this approach stands to help us understand how representations of the same factors shift from one area to another, and how shifts in the trial-by-trial activity in an upstream area produce better or worse representations in a downstream area. It also provides a way to look at how different areas reshape the same information in order to “untangle” it, or increase the linear separability of a biologically relevant variable (Figure 2D; DiCarlo and Cox, 2007; DiCarlo et al., 2012; Pagan et al., 2013). In future work, dimensionality reduction may be combined with data conditioning in order to determine how the representation in a recipient area depends on the state of a simultaneously recorded source area.

This problem extends to reasoning about how different areas contribute to different aspects of a complex task. Yates et al. (2017) combined measurements of behavior and the spike-count correlations within and between areas MT and LIP, with models of the two areas. They were able to dissect a perceptual decision-making task into several components that are partially shared between MT and LIP, but did not find any evidence of single-trial coupling between these two areas, which is inconsistent with theories that LIP integrates the information in MT. Simultaneous population recordings in multiple areas alone permit this kind of trial-by-trial assessment of how information is transferred and transformed, and will be useful for separating hierarchical computations from computations that are apparent at many stages of the hierarchy.

### How Do Global Factors Modulate Inter-Area Cortico-Cortical Communication?

Variability in the responses of neurons, as measured with spike-count correlations, can be partly explained by modulating factors such as anesthetic state, attention, and arousal (Goris et al., 2014; Rabinowitz et al., 2015). It is unclear how these “global” factors interact with local factors (such as adaptation or stimulus context), and what the scale of the modulations induced by these global factors truly is. By recording population activity in multiple areas, we will be able to determine the scope of local and global factors, for example, to determine how far local network changes propagate through the cortical hierarchy. Sub-cortical systems play a significant role in modulating cortical processing (Sherman, 2016). Expanding simultaneous multi-area cortical recordings to include related subcortical systems, potentially in small brains with large, multi-contact probes (Jun et al., 2017), may be profoundly informative for learning why cortical states tend to shift, both “spontaneously” and in a task-dependent way (Ruff and Cohen, 2018).

## Conclusion

We are able to measure larger populations than ever, but characterizing many predicted theoretical effects requires recording from exceedingly large-scale populations (hundreds or thousands of neurons). While most electrophysiology is currently constrained to monitoring hundreds of neurons, imaging approaches are able to monitor thousands but have poor temporal resolution. Improved temporal resolution of imaging and higher-yield electrophysiology experiments will move the field forward substantially.

Population size aside, dimensionality reduction requires repeating each trial a large number of times (and indeed, the number of necessary repetitions increases with the number of cells simultaneously recorded). The recording stability required for these measurements can be difficult to obtain in an anesthetized preparation and the timescale is potentially impossible in awake animals until recordings can be reconciled with carefully quantified natural behaviors. In single-area recordings, the limits of the anesthetized preparation are reasonably well-understood, but it is not yet clear if inter-area dynamics are as consistent as basic sensory representations between the anesthetized and awake states. Modest increases in population size, along with the technological advances that permit us to characterize each cell more completely (e.g., laminar profile, genetic markers, morphology, receptive field substructures, connectivity) will let us make stronger inferences about the varied roles different cells play in shaping population activity, and thus perception, cognition, and behavior.

## Author Contributions

EZ and NP wrote and edited the article.

## Conflict of Interest Statement

The authors declare that the research was conducted in the absence of any commercial or financial relationships that could be construed as a potential conflict of interest.
